# Clinical Skills Day: A Novel Approach to Enhancing Procedural Skills Teaching for Foundation Year One Doctors

**DOI:** 10.7759/cureus.31276

**Published:** 2022-11-08

**Authors:** Alexis Adam, Anisha Mangtani, Chris Jacobs, Jessica Daniel

**Affiliations:** 1 Postgraduate Medical Education, Great Western Hospitals National Health Service (NHS) Foundation Trust, Swindon, GBR

**Keywords:** foundation year one doctor, procedural skills training, teaching, clinical skills day, medical education, uk foundation programme

## Abstract

Background

Completion of the Foundation Year One (FY1) doctor training is a requirement for full General Medical Council registration in the United Kingdom. Training during this year is mapped to a curriculum with one of the key elements being safe procedural skills. The objective of this project was to improve the teaching of procedural skills through the means of a Clinical Skills Day (CSD) and to quantify any improvement.

Materials and methods

A one-group pretest-posttest design was conducted on 32 doctors who completed a confidence inventory before and after four core procedural stations: suturing, urethral catheterisation (both male and female), lumbar puncture, and bimanual and speculum examinations. The intervention of simulated procedural skills occurred under the supervision of senior clinicians, with FY1 doctors receiving teaching and practising the four skills. The primary outcome was the impact of a CSD on trainees’ confidence in performing certain skills. Pre- and post-CSD trainees’ confidence levels were collected via an online-focused questionnaire and descriptive statistics, paired t-test, and one-way analysis of variance (ANOVA) with post-hoc Bonferroni comparisons were undertaken for statistical analysis.

Results

The difference in the mean scores of confidence post-intervention was significant in all four procedural stations with or without supervision (p <0.0001).

Conclusions

The use of CSDs impacted positively on the FY1 doctors’ confidence in performing certain skills. Wider implementation of this promising approach for Foundation Doctors is recommended.

## Introduction

United Kingdom (UK) Foundation Programme and its curriculum

Following graduation from medical school, newly qualified doctors embark on a career-long journey where learning and professional development are paramount. In the UK, the first step in that respect consists of joining and completing the UK Foundation Programme [1,2]. This two-year-long programme aims to develop and enhance the clinical knowledge and skills of medical graduates, to learn about the UK healthcare environment and explore different career paths [3]. Foundation Year One (FY1) doctors (doctors in their first year of training) and Foundation Year Two (FY2) doctors (doctors in their second year of training) need to demonstrate that they are competent in several areas, such as clinical care and professionalism, to successfully complete the UK Foundation Programme and continue their journey in specialty training. Professional development during this stage of training is aligned with a defined curriculum. The latter, which was reviewed and reformed in 2021, comprises three Higher Level Outcomes (HLOs) [4]: HLO 1 - An accountable, capable and compassionate doctor; HLO 2 - A valuable member of the healthcare workforce; and HLO 3 - A professional responsible for their own practice and portfolio development.

Barriers to clinical skills teaching for Foundation Doctors

The ability of the Foundation Doctors to perform different procedural skills confidently and accurately is a key element of the UK Foundation Programme curriculum and is encompassed in HLO 1 [4]. The importance of knowledge, skills, and performance is furthermore emphasised in the Good Medical Practice guideline from the UK medical licensing body, the General Medical Council [5]. However, learning procedural skills in a clinical environment can be challenging for doctors in training. Several barriers have been faced by trainees such as lack of time, insufficient clinical cover, and lack of motivation [6]. Additionally, some procedural skills, such as lumbar puncture, have been reported as being more complicated for junior trainees and low levels of confidence have been elicited [7].

The proposed solution was the introduction of a Clinical Skills Day (CSD) for Foundation Doctors, which consists of a dedicated day (five hours of training) where the trainees practise certain procedural skills in a simulated environment under the guidance and supervision of more experienced clinicians. CSDs have already been integrated into the UK undergraduate and post-graduate settings; for example, FY2 doctors working in the Southwest of England must attend a regional CSD in order to satisfy the requirements for completion of their training [8]. However, a literature review highlighted a lack of evaluation of CSD in the context of FY1 doctors’ training. Additionally, CSD does not constitute a curriculum requirement at the FY1 level.

Research questions

The following research questions were used: 1. Do CSDs increase the confidence of FY1 doctors in performing selected procedural skills? 2. Does the extent of the impact, if any, of CSDs on FY1 doctors’ confidence vary between selected procedural skills?

## Materials and methods

Design and setting

We conducted a one-group pretest-posttest design on FY1 doctors who completed a confidence inventory before and after four selected procedural skills stations as part of our CSD. 

The first step in designing the CSD was to decide which skills warranted inclusion in this training session. After discussion with the academic team of the postgraduate medical education department, the four skills selected were: suturing, urethral catheterisation (both male and female), lumbar puncture, and bimanual and speculum examinations. Following this, the requisite equipment and clinical models were ordered, and the help of senior clinicians was sought to volunteer as facilitators. 

**Figure 1:**
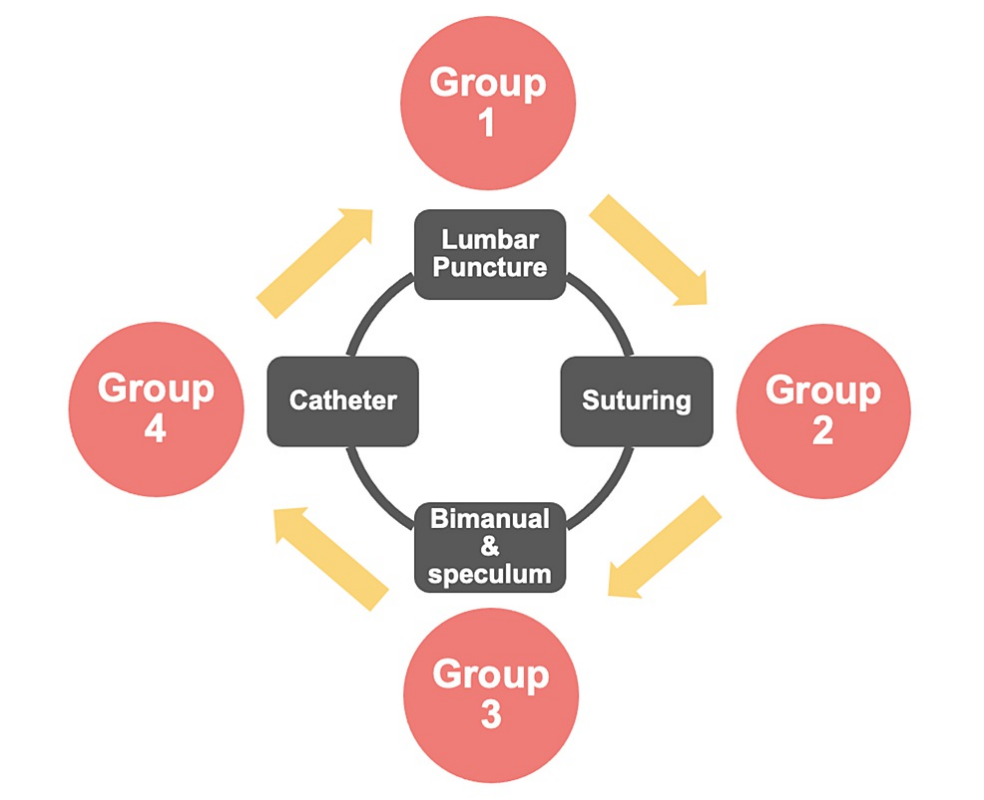
Schematisation of the Foundation Year One (FY1) Clinical Skills Day for cohorts A and B

For logistical reasons, the targeted group of FY1 doctors were divided into two cohorts and attended the CSD on two different dates, with different facilitators present on each date. On each day, the cohort of FY1 doctors was further divided into four groups, rotating through the above-mentioned procedural skills sessions (Figure 1). During each session, the FY1 doctors received information and explanation concerning a specific clinical procedure before being demonstrated the skills by one of the facilitators. FY1 doctors then had the opportunity to practice the procedure in a simulated setting using the various clinical models and equipment ordered. 

Data collecting tool

**Figure 2:**
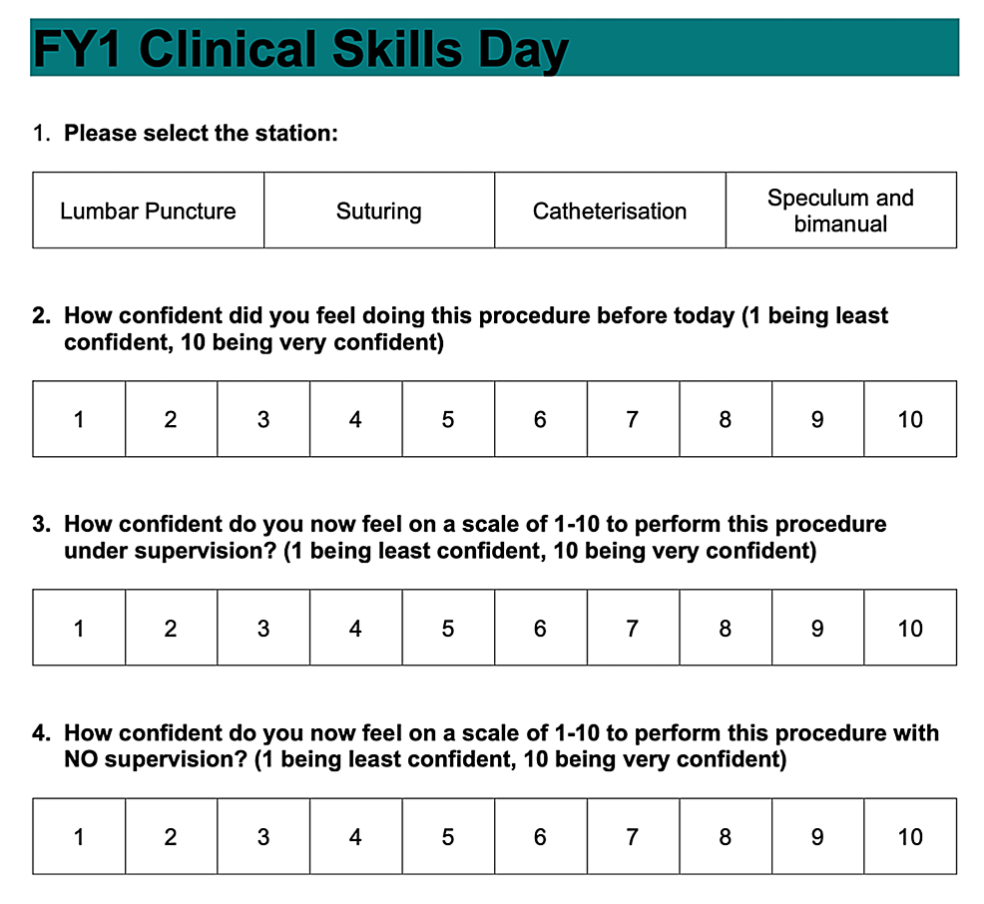
Format of the online survey used for data collection FY1 = Foundation Year One

The primary outcome measure was whether the confidence of FY1 doctors regarding the four above-mentioned procedural skills improved following attendance of the FY1 CSD. A quick response (QR) code linked to an online-focused questionnaire was placed at the four clinical skills stations (Figure 2). FY1 doctors were encouraged by the facilitators to complete the questionnaire before rotating to the next station. The online-focused questionnaire comprises three Likert-type scales to assess trainees’ confidence regarding a specific skill [9,10]: Question 1 - Assessment of trainees’ confidence pre-CSD. Question 2 - Assessment of trainees’ confidence post-CSD supervised. Question 3 - Assessment of trainees’ confidence post-CSD unsupervised. MS Excel version 2010 was used to maintain the generated database. 

Participants

In this research, all the 42 FY1 doctors working at Great Western Hospitals National Health Service (NHS) Trust, Swindon, UK, from August 2021 to August 2022 formed the population. The following inclusion criteria were applied and only those fully satisfying them were included in this study: 1. Working as an FY1 doctor at Great Western Hospitals NHS Trust from August 2021 to August 2022; 2. Attended the FY1 CSD; and 3. Completed the online-focused questionnaire during the FY1 CSD.

**Figure 3:**
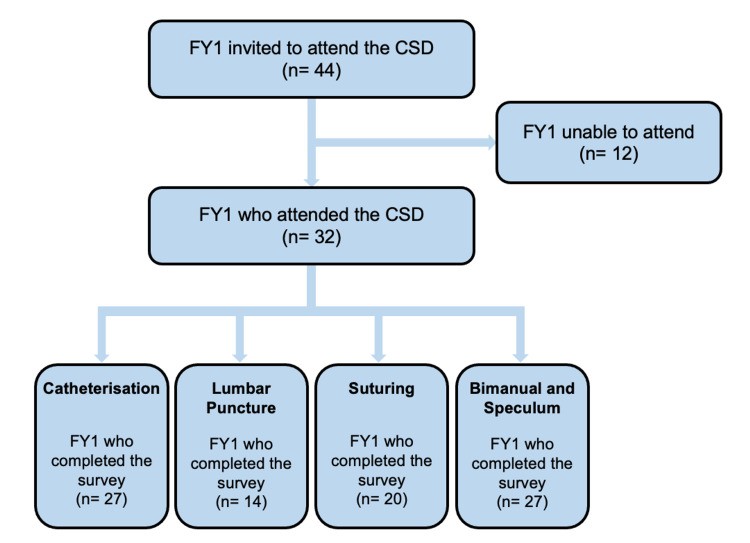
Schematic representation of the participation rate and focused questionnaire completion rates for this study FY1 = Foundation Year One; CSD = Clinical Skills Day

Out of the 44 FY1 doctors who were invited to the CSD, 32 attended (72.7%). Non-attendance was explained by FY1 doctors as having other clinical commitments or being on leave. Online questionnaire completion rates varied significantly across the four stations with an average of 68.8% (Figure 3). The reason for this variation in completion rates is unclear, although it could potentially be explained by facilitators at different stations having different approaches in reminding the FY1 doctors to complete the focused questionnaire. 

Statistical analysis plan

A retrospective analysis of the outputted data examining the three elements of the primary outcome measure (trainees’ confidence pre-CSD, post-CSD under supervision, and post-CSD without supervision) was performed. The analysis focussed on quantifying the improvement, if any, of the confidence of the FY1 doctors for the specific skills covered during the FY1 CSD. 

A numerical and graphical descriptive statistical analysis was initially performed using MS Excel version 2010. The data was subsequently scrutinised further using inferential statistical analyses. Paired t-test and one-way analysis of variance (ANOVA) with post-hoc Bonferroni comparisons were undertaken using StatsDirect version 3.3.5 (Released 2021; StatsDirect Ltd, Birkenhead, UK). 

Ethics

This study was supported by the Postgraduate Medical Education Department at Great Western Hospitals NHS Trust. NHS Research Ethics Committee review was not required as per UK NHS Health Research Authority guidelines [11]. 

## Results

Descriptive statistics and preliminary analyses

**Figure 4:**
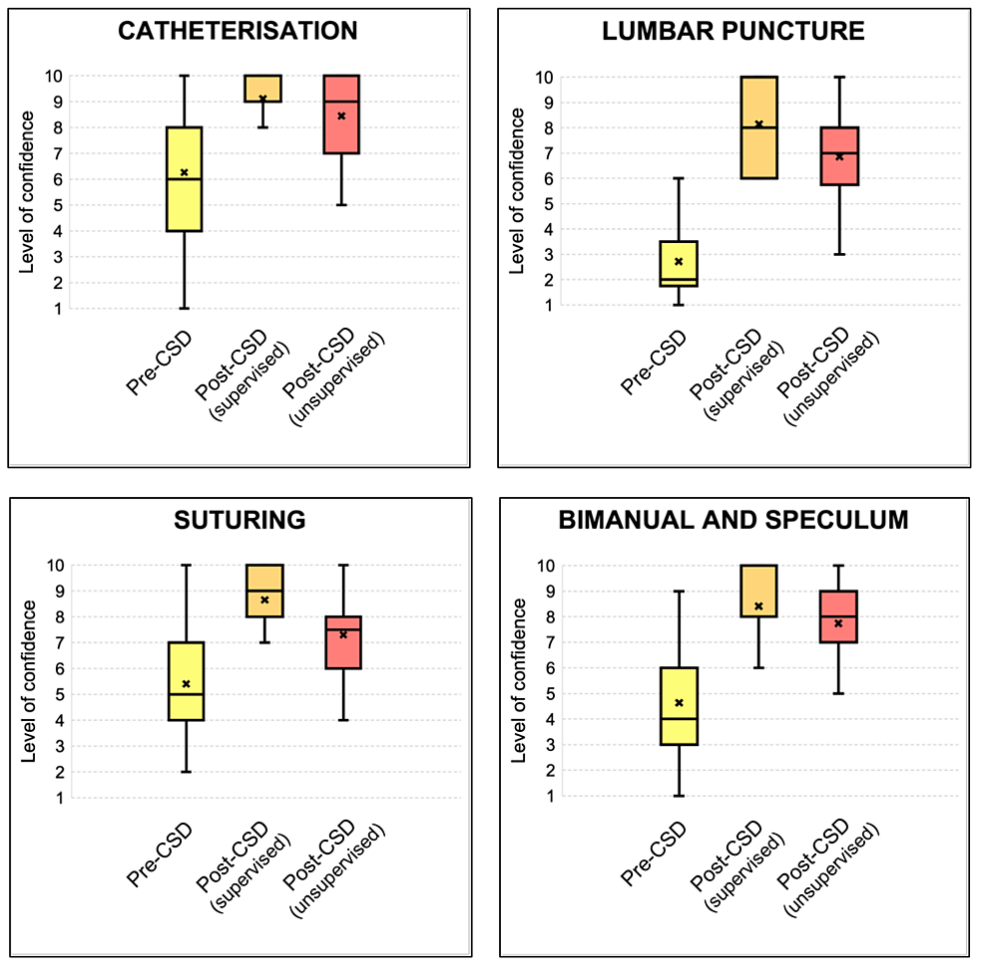
Box plots presenting the level of confidence for different procedural skills pre- and post-CSD CSD = Clinical Skills Day

Before the FY1 CSD, the average confidence in performing the four procedural skills included in this CSD averaged 4.8 out of 10. Catheterisation scored the highest pre-CSD with an average of 6.3 out of 10, followed by suturing with 5.4 out of 10, and bimanual and speculum examination with 4.6 out of 10. Lumbar puncture scored the least with 2.7 out of 10 (Table 1; Figure 4). An increase in FY1 doctors’ confidence is noted across the four stations post-CSD for both supervised and unsupervised activity, although this is less markedly evidenced when considering the latter. The average post-CSD confidence score when carrying the four procedural skills under supervision rose to 8.6 out of 10, which translates into a 79.2% increase from pre-CSD scores. The most notable increase was seen with lumbar puncture with the average confidence score increasing to 8.1 out of 10. When considering the average confidence score for unsupervised activity post-CSD, a 58.3% increase is noted, bringing the average confidence score to 7.6 out of 10. 

**Table 1:** Table presenting the mean level of confidence for different procedural skills pre- and post-CSD FY1 = Foundation Year One; CSD = Clinical Skills Day

	Mean level of FY1 doctors’ confidence		
Skills	Pre-CSD	Post-CSD (supervised)	Post-CSD (unsupervised)
Catheterisation	6.3	9.1	8.4
Lumbar puncture	2.7	8.1	6.9
Suturing	5.4	8.7	7.3
Bimanual and speculum	4.6	8.4	7.7

Main analysis

Research Question 1

Paired t-test results of pre- and post-test confidence scores following intervention revealed a significant improvement (p < 0.0001). The post-CSD score change was greatest under supervision (mean difference -3.64, 95% CI -4.15 to -3.12). Participants reported a statistically significant improvement under no supervision (mean difference -2.72, 95% CI -3.18 to -2.26). 

Research Question 2

Normality checks were carried out with data approximating a normal distribution. Repeated measures of within-subject design analysed by one-way ANOVA indicated that the improvements in four CSD scores differed (F (3,84) =4.78 p=0.004). Furthermore, post-hoc Bonferroni comparisons identified lumbar puncture as the most significant in confidence change with and without supervision (p<0.05).

## Discussion

The above results reveal significant improvement in the confidence of FY1s with procedural skills after attending a CSD. By offering a safe and supported environment for the practice of certain skills, CSD positively impacted the trainees’ procedural skills. 

Some of the skills, such as lumbar puncture, recorded poor levels of confidence pre-CSD. This could reflect the greater complexity of the tasks and the lack of junior doctors’ opportunity to practise them in a clinical environment [12]. Significant improvement in the trainees’ confidence post-CSD was however elicited, highlighting further the valuable impact of CSD. 

Another noteworthy finding is the decline in the trainees’ confidence post-CSD when no supervision is provided compared to when supervision is. Although FY1 doctors are at an early stage of their postgraduate medical training, striving to guide trainees to become safe and independent practitioners is paramount. More support and guidance during the CSD could potentially achieve this. 

Recommendations and limitations

Given the positive outcome of the FY1 CSD, its wider implementation is recommended. Integration of the CSD as a key element of the FY1 doctors’ training should be considered, although the authors acknowledge that organisational barriers faced by FY1 doctors, such as the study leave allowance, need to be explored and addressed. 

Nevertheless, despite the optimistic perspective that this study offers when it comes to enhancing procedural skills teaching for FY1 doctors, careful adoption and generalisation of the findings are required. Firstly, this study focuses solely on the evaluation of the confidence of the FY1 doctors pre- and post-CSD. Although it has been assumed that an increased level of confidence would positively correlate with an increased level of performance, some studies suggest it might not be the case and more objective data should therefore be considered [13]. Pre- and post-CSD formative assessment of the trainees’ performances by more senior clinicians could allow this. 

Additionally, this study does not take into consideration the long-term impact of CSD. The confidence of trainees is at risk of declining unless continuous reinforcement of the taught skills is implemented [14]. Lastly, other teaching mediums have been described in the literature and should be studied and examined against CSD with a view to optimising procedural skills teaching for FY1 doctors in the UK [15]. 

## Conclusions

The use of CSD impacted positively the FY1 doctors’ confidence in performing certain skills. Additionally, some of the selected skills that have been reported as more challenging for FY1 doctors, such as lumbar puncture, recorded a significant increase in the trainees’ level of confidence, highlighting further the valuable impact of CSD. Wider implementation of this promising approach to enhancing procedural skills teaching for FY1 doctors is recommended. However, the need for further studies exploring CSD and its long-term impacts is recognised.
